# **Information needs and information seeking behaviour of patients during follow-up of colorectal cancer in the Netherlands**

**DOI:** 10.1007/s11764-019-00779-5

**Published:** 2019-07-08

**Authors:** T. Wieldraaijer, L. A. M. Duineveld, W. A. Bemelman, H. C. P. M. van Weert, J. Wind

**Affiliations:** 1 Department of Primary Care, Amsterdam UMC, University of Amsterdam, location AMC, 22660, 1100 DD Amsterdam, the Netherlands; 2 Department of Surgery, Amsterdam UMC, location AMC, Amsterdam, the Netherlands

**Keywords:** Colorectal cancer, Follow-up, General practitioners, Patients, Information needs, Information seeking behaviour

## Abstract

**Purpose:**

Adequately informing patients is considered crucial in cancer care, but need for information and information seeking behaviour of colorectal cancer (CRC) patients in the Netherlands are currently not well known.

**Methods:**

In a prospective study, patients participating in a specialty, hospital-based follow-up program completed three consecutive surveys over a 6-month period to analyse their information need and information seeking behaviour.

**Results:**

Patients (*n* = 259) felt well informed about their treatment (86%), disease (84%), and follow-up program (80%), but less well informed about future expectations (49%), nutrition (43%), recommended physical activity (42%), and heredity of cancer (40%). The need for more information on these subjects remained constant over the first five postoperative years. Patients who were younger, who had undergone chemotherapy, or who had comorbid conditions needed more information on several subjects. One in three patients searched for information themselves, mostly on the Internet. One in four patients consulted a health care provider for information, mostly their GP. Younger and more educated patients more often searched for information themselves, while patients undergoing chemotherapy more often consulted the hospital nurse. Information seeking behaviour remained constant over time.

**Conclusions:**

This study showed where current information provision is perceived as adequate and on which subject improvements can be made. It identifies information seeking behaviour and proposes ways to personalize information provision.

**Implications for Cancer Survivors:**

The GP is most frequently consulted for information; involving GPs in CRC follow-up could improve information provision on several subjects for several patients.

## Introduction

Providing information that patients need is considered crucial for adequate cancer care [1, 2]. Patients who are better informed have a higher health-related quality of life, and less anxiety and depression [3–5]. The need for information varies between types of cancer and sometimes evolves as patients pass through different stages of their cancer care continuum [6, 7]. After treatment with curative intent, colorectal cancer (CRC) patients in the Netherlands are included in a follow-up program for 5 years to detect possible recurrent disease and guide them through the years following treatment [8]. During this period, patients are able to ask questions and kept informed about relevant health issues. Traditionally, this follow-up for CRC patients is performed mainly in secondary care, but there is an increasing tendency to involve general practitioners (GPs) more during this stage of care [9–12].

Previous research has been performed on the information needs or information seeking behaviour of patients after initial treatment for CRC [6, 13]. These patients in the USA were found to be well informed about their disease and treatment, but less well informed about reducing the risk of cancer recurrence, improvements in lifestyle, and the potential benefit of involving a primary care provider in their care. Other subjects that were identified to receive less attention during follow-up were diet/nutrition and managing bowel symptoms [7]. Interestingly, in only a minority of the articles, patients had actually been *asked* about their needs, and few articles reported *how* patients searched for information [6, 7]. Many studies published on the subject are presented on the online database of the US Health Information National Trends Survey [14]. Finney Rutten et al. demonstrated that information seeking is common in cancer survivors, and the percentage of survivors seeking information is increasing in recent years [15].

The information needs and information seeking behaviour of CRC patients in the Netherlands are currently not well known. The regular follow-up visits take place at the hospital, while every patient in the Netherlands also has a regular GP who acts as first contact and often provides continuous care. Patients might therefore consult several health care providers for information. To what extent patients feel informed by health care providers and whether they look for information themselves needs to be examined to see if improvements can be made.

Therefore, we performed a prospective study in which all patients currently in a CRC follow-up program were asked to complete three consecutive surveys. In this paper, we aim to report on (1) whether patients feel informed about the different subjects of follow-up care and if they need more information on any subject, (2) whether and how patients look for information themselves, and (3) whether there are any differences in subgroups of patients.

## Methods

### Patients

We performed a prospective cohort study among patients treated with curative intent for CRC and who were currently participating in a follow-up program (i.e. the first five postoperative years). Recruitment was done at the outpatient clinics of six Dutch hospitals. Patients with TNM stages 1–3 disease were included. Patients were eligible if they had a (temporary) stoma, and if they had received or were still undergoing adjuvant chemotherapy. Patients were excluded in case of hereditary CRC, inflammatory bowel disease, (sub)total colectomy, history of other primary cancer, or any other condition where specialized follow-up care was needed. We identified patients by means of hospital databases used for follow-up planning.

### Survey

Patients were asked to complete an identical survey at three different times: (1) at inclusion of the study, (2) 2 months after inclusion, and (3) 6 months after inclusion. This survey contained questions on sociodemographic background, lifestyle, information provision, information need, and information searching. Information about cancer characteristics, staging, treatment, comorbid conditions, and medication use was obtained from patients’ hospital and general practice records.

### Statistics

The data were collected using an online survey program (SurveyMonkey) and analysed using SPSS Statistics 25 and MLwiN 2.34. We performed an independent samples *t* test and chi-squared test for comparison between participants and non-participants. To examine the differences between specific subgroups of patients, we performed a mixed effects logistic regression to account for the repeated observations within patients. Thematic analysis was performed for responses to open questions. When presenting the results over time, we divided all patients into three groups, according to their time after surgery at the time of filling out the survey: (1) within 6 months after surgery, (2) between 6 and 12 months after surgery, and (3) later than 12 months after surgery. We chose this way of representation because analyses of longitudinal data from individual participants and subgroups of patients did not show any change over time. We confirmed the validity of dividing the patients in this way, and checked that changing the number of groups or the time frames used did not change the outcomes presented here.

### Ethical statement

The Medical Ethics Committee of the Amsterdam University Medical Centres reviewed the protocol and judged that a formal evaluation by the committee was not required. Nevertheless, all participants received study information and provided verbal and written consent.

## Results

Four hundred eighty-two patients were contacted of whom 259 agreed to participate (response rate 54%); 222 patients completed all three surveys (86%). Patients that did not participate were older (average 72 versus 67 years, *p* < 0.001) but otherwise similar to the participating group. Reasons for not participating were as follows: too much effort (*N* = 66), did not wish to disclose a reason (*N* = 47), lack of interest (*N* = 41), the study being too confrontational (*N* = 36), feeling too old/feeble (*N* = 20), or other reasons (*N* = 13).

Table 1 shows the characteristics of all participants. The average age was 67 years (range 32–94), 54% were male, and the median time after surgery at baseline was 7 months (range 0–60). One in three participants had undergone adjuvant chemotherapy, with a quarter of those (*n* = 35) undergoing chemotherapy during the survey. Fifty percent of all patients had one or more chronic comorbid condition.

**Table 1:** Patient and disease characteristics at baseline

	Patients (*N* = 259)	
Age (mean years, SD^1^)	67	(SD 10.1)
Male (%)	141	(54%)
Time after surgery (median in months, IQR^2^)	7	(4–13)
Tumour stage^3^		
1 (%)	73	(28%)
2 (%)	88	(34%)
3 (%)	98	(38%)
Location of tumour		
Colon	230	(89%)
Rectum	29	(11%)
Type of surgery		
Right hemicolectomy	116	(45%)
Transverse colectomy	6	(2%)
Left hemicolectomy	21	(8%)
Sigmoid colectomy	55	(21%)
Recto-sigmoid resection	56	(22%)
Abdominoperineal resection	5	(2%)
Chemotherapy		
Currently undergoing adjuvant chemotherapy	35	(14%)
Finished adjuvant chemotherapy or neo-adjuvant chemoradiotherapy^4^	59	(23%)
Living situation		
Living together	198	(76%)
Living alone	61	(24%)
Employment status		
Active	46	(18%)
Sick leave	21	(8%)
Retired	192	(74%)
Educational attainment		
Primary or none	12	(5%)
Secondary	149	(58%)
Vocational education	70	(27%)
University	28	(11%)
Chronic comorbid condition		
None	129	(50%)
Cardiovascular disease	88	(34%)
Diabetes mellitus	31	(12%)
Severe arthrosis	25	(10%)
Asthma/COPD	18	(7%)
Depression	14	(5%)
Other^5^	41	(16%)
Stoma	38	(15%)
Smoking		
Yes	25	(10%)
Previously	151	(58%)
No	83	(32%)
Medication use		
Prescribed medication	115	(44%)
Over-the-counter medication only	67	(26%)
None	77	(30%)
Regular physical activity		
None	70	(27%)
Low intensity (such as walking, cycling)	93	(36%)
High intensity (such as sports, exercise)	96	(37%)

^1^*SD* standard deviation

^2^*IQR* interquartile range

^3^Tumour stage was defined using the TNM5 criteria

^4^Given only in rectal carcinoma

^5^Reported by less than 5% of respondents; renal failure, liver disease, skin disease, peptic ulcers, and various other disorders

Table 2 shows the frequency of health care provider visits reported by patients. Two thirds of patients consulted their GP 1–5 times the previous year, with a small percentage (6%) consulting their GP more than 10 times. This consultation rate remained constant over time. Most patients had a regular surgeon or oncology specialist, and about half of patients had a regular specialized hospital nurse. Most patients reported to feel reassured by their surgeon or oncology specialist and specialized hospital nurse, and most patients reported being involved in making their own treatment decisions (Table 2).

**Table 2:** Self-reported experience with health care providers

	< 6 months after treatment (*N* = 187)		6–12 months after treatment (*N* = 239)		> 1 year after treatment (*N* = 198)	
How many times have you visited your GP in the last 12 months?	N	(%)	N	(%)	N	(%)
Not once	11	(6)	16	(7)	33	(17)
1–5 times	129	(69)	165	(69)	128	(65)
5–10 times	35	(19)	42	(18)	25	(13)
> 10 times	12	(6)	16	(7)	12	(6)
Question	Yes	(%)	Yes	(%)	Yes	(%)
Do you have a regular specialist at the hospital?	162	(87)	216	(90)	176	(89)
Is this important to you?	170	(91)	217	(91)	181	(91)
Do you have a regular nurse at the hospital?	97	(52)	112	(47)	70	(35)
Is this important to you?	120	(64)	133	(56)	98	(50)
Do you know how to contact other caregivers when necessary (such as social worker, psychologist, nutritionist, and physical therapist)	169	(90)	216	(90)	169	(85)
Is this important to you?	154	(82)	189	(79)	165	(83)
Were you involved in deciding you own treatment?	158	(85)	193	(81)	163	(82)
Is this important to you?	97	(52)	205	(86)	174	(88)
Does your surgeon/oncology specialist reassure you?	176	(94)	229	(96)	188	(95)
Is this important to you?	183	(98)	236	(99)	192	(97)
Does your hospital nurse reassure you?	155	(83)	202	(85)	158	(80)
Is this important to you?	160	(86)	202	(85)	162	(82)

**Fig. 1:**
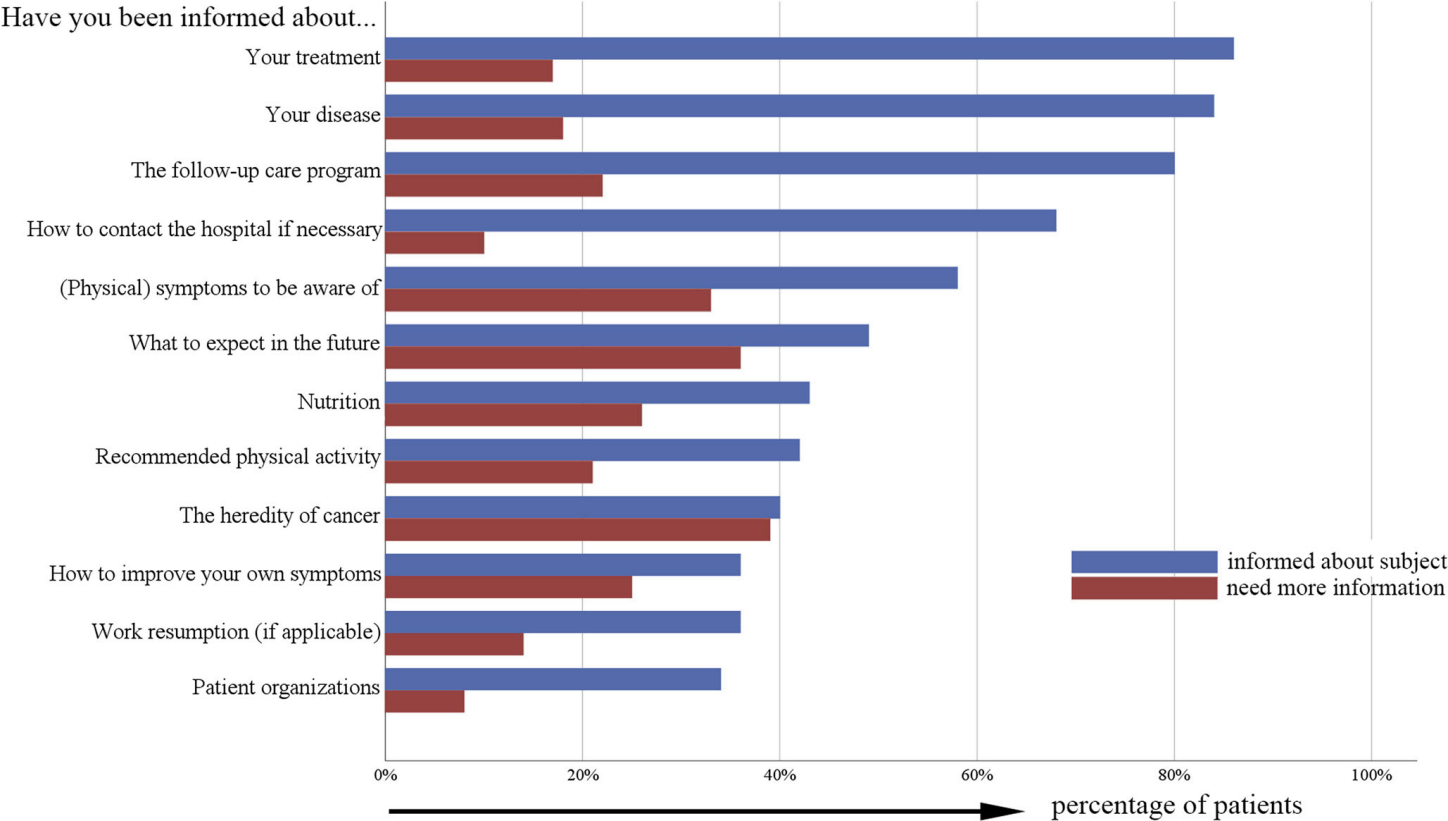
Number of patients who reported feeling informed about various subjects, and whether they thought they needed more information on these subjects

Figure 1 shows how many patients reported feeling informed about various subjects, and whether they thought they needed more information on these subjects. Patients who recently (< 6 months) had surgery felt more informed about their treatment (odds ratio (OR) 2.17, 95% confidence interval 1.25–3.76), and disease (OR 2.17, 95% CI 1.28–3.65), but no other differences over time were found on *any* of the subjects or needs. Patients felt most informed about their treatment (86%), disease (84%), follow-up program (80%), and how to contact the hospital if necessary (68%). Need for more information on these subjects was low. Patients felt less informed about what to expect in the future (49%), nutrition (43%), recommended physical activity (42%), the heredity of cancer (40%), and how to improve their symptoms (36%). Need for more information was especially high on the heredity of cancer (39%), what to expect in the future (36%), and nutrition (26%) (Fig. 1).

To examine the need for more information on lifestyle, patients responded to several statements. About half of the patients reported that cancer had made them eat healthier, or intended to eat (even) healthier. Only a small percentage needed guidance to eat healthier. About one in five patients had become physically more active because of their cancer, and one in three intended to get more active. The need for guidance to get more active was low. The cancer diagnosis had made two thirds of patients stop smoking, but only between 6 and 12 months after treatment. The intention to stop smoking and the need for help to stop smoking were low.

Patients younger than 65 years felt more informed about the heredity of cancer (OR 2.04, 95% CI 1.39–3.00), and *needed* more information than older patients on the follow-up program (OR 1.61, 95% CI 1.06–2.46), what to expect in the future (OR 1.57, 95% CI 1.06–2.31), nutrition (OR 1.53, 95% CI 1.00–2.34), and ways to improve their symptoms (OR 1.79, 95% CI 1.18–2.71). Patients that had undergone chemotherapy or were currently undergoing chemotherapy felt more informed about (physical) symptoms to be aware of (OR 1.54, 95% CI 1.05–2.25), what to expect in the future (OR 1.60, 95% CI 1.09–2.33), nutrition (OR 1.48, 95% CI 1.02–2.13), recommended physical activity (OR 1.71, 95% CI 1.18–2.48), ways to improve their (physical/mental) symptoms (OR 1.79, 95% CI 1.24–2.57), but also *needed* more information on their treatment (OR 1.69, 95% CI 1.04–2.73), and follow-up program (OR 1.90, 95% CI 1.25–2.89). Patients with a chronic comorbid condition needed more information on how to contact the hospital if necessary (OR 2.46, 95% CI 1.23–4.90), what to expect in the future (OR 1.55, 95% CI 1.06–2.25), recommended physical activity (OR 1.74, 95% CI 1.10–2.75), and the heredity of cancer (OR 1.81, 95% CI 1.26–2.61).

Table 3 shows information seeking behaviour over time. One in three patients reported searching for information themselves, mostly on the Internet (65%), by asking friends (28%), or by reading an information brochure (25%). Patients younger than 65 years more frequently searched for information themselves (OR 1.66, 95% CI 1.13–2.43), and more often used the Internet (OR 2.58, 95% CI 1.66–3.99). Patients with a university education also more frequently searched for information themselves (OR 2.18, 95% CI 1.24–3.82), and used the Internet (OR 3.08, 95% CI 1.69–5.62) and friends (OR 3.26, 95% CI 1.58–6.72) more frequently as sources. One in four patients consulted one or more health care providers for information, mostly their GP (50%), surgeon or oncology specialist (45%), or hospital nurse (26%). Patients currently undergoing chemotherapy were more likely to consult their specialized hospital nurse for information (OR 3.30, 95% CI 1.33–8.21). Information seeking behaviour and sources of information remained constant over time.

**Table 3:** Information seeking behaviour over time

	< 6 months after treatment (*N* = 187)		6–12 months after treatment (*N* = 239)		> 1 year after treatment (*N* = 198)	
	Yes	(%)	Yes	(%)	Yes	(%)
Have you searched for information yourself?	64	(34)	77	(32)	66	(33)
If yes, where?						
1. Internet	44	(69)	44	(57)	47	(71)
2. Friends	21	(33)	20	(26)	16	(24)
3. Information brochure	18	(28)	18	(23)	16	(24)
Other miscellaneous^†^	9	(14)	10	(13)	8	(12)
Have you consulted a caregiver for additional information?^‡^	42	(23)	59	(25)	47	(24)
If yes, whom?						
1. GP	18	(43)	30	(51)	26	(55)
2. Surgeon/oncology specialist	12	(29)	23	(39)	32	(68)
3. Hospital nurse	17	(41)	10	(17)	12	(26)
Other miscellaneous^§^	28	(67)	58	(98)	36	(77)

^†^Reported by less than 5% of patients: patient organization (2%), colleagues (1%), books/magazines (1%)

^‡^More than one caregiver could be reported; numbers overlap

^§^Reported by less than 5% of patients: physical therapist (4%), alternative medicine practitioner (3%), specialist other than follow-up care coordinator (3%), assistant to GP (3%), pharmacist (2%), psychologist (2%), nutritionist (1%), social worker (1%)

## Discussion

This paper reports on the information needs and information seeking behaviour of colorectal cancer (CRC) patients after initial treatment. By means of three consecutive surveys, we prospectively evaluated (1) whether patients felt informed about the different subjects of follow-up and if they needed more information on any subject, (2) whether and how patients looked for information themselves, and (3) whether there were any differences in subgroups of patients.

### Main findings

CRC patients in the current health care situation feel well informed about their treatment, disease, follow-up program, and how to contact the hospital if necessary. Other subjects, such as future expectations, nutrition, recommended physical activity, and heredity of cancer are perceived to be less well addressed. Especially high was the need for more information on heredity and what to expect in the future. Patients felt least informed about work resumption and patient organizations, but the need for more information on these subjects was also low. Apart from patients that have recently undergone surgery being more informed about their treatment and disease, there were no significant changes over time for any of the subjects or needs described in this study.

One in three patients in our study searched for information themselves, mostly by using the Internet. One in four patients reported visiting a health care provider specifically for more information, most frequently the GP. The information seeking behaviour did not change with time after surgery.

Younger patients and patients with a university education were more informed, and more frequently looked for information themselves. Patients who had undergone chemotherapy were also more informed about several subjects, but also needed more information on their treatment and follow-up program. Patients with chronic comorbid conditions needed more information on several subjects, but did *not* search for more information themselves, or consult a health care provider for more information.

### Comparison to previous literature

The subjects on which the patients in this study felt well informed are in line with previous reports [6, 7, 13, 15]. The provision of information could be improved most where patients’ unmet need for information is highest. As concluded by Salz et al., it would appear that CRC patients need more information that allows them to plan for the future, as opposed to more information on the treatment they have undergone [13]. For instance, more information on nutrition, recommended physical activity, and ways to improve long-term physical and mental symptoms could possibly serve these needs [4, 7, 13, 16–19]. Information on the heredity of cancer appeared to be the most prominent unmet need in our patients. CRC follow-up care providers should take note of this need for information on heredity and address the issue, even if they think heredity does not apply to the medical situation of their patient. The patients in our study reported a low need for information on work resumption even after correction for working status, which differs from previous reports [19]. Possibly, this difference is due to the different cancer types included in other studies (such as breast cancer and melanoma) with a lower average age of patients, whereas our study population consisted mainly of older patients, who are more at the end of their working lives.

Previous studies have shown that CRC patients that have received dietary advice were more likely to change their diet, although lifestyle issued are not always adequately addressed by health care providers [20, 21]. Tan et al. state that “clinicians could consider addressing issues including smoking cessation, physical activity, or other risk behaviours with their patients during the early survivorship period when patients are likely to be more receptive to information about managing risks of recurrence” [6]. However, the low percentage of patients in our study that intend to improve their lifestyle or need guidance in doing so indicates that these subjects are not something they are keen on addressing. And yet, combined with the unmet need on information about nutrition in particular, this observation could be a starting point to improve information on lifestyle that could contribute to an improved lifestyle and higher quality of life in CRC patients, certainly in the long run.

Previous studies vary widely in the reported use of the Internet [7, 22–24], but this probably has to do with differences in study populations and the increasing familiarity with the Internet as a medium over time. More important is a concern expressed by Cumbo et al. and Sajid et al. about the lack of quality control on the information found on the Internet [22, 23]. Anticipating this concern, there have been efforts to provide cancer patients with reliable and accessible Internet-based sources of information that are currently being evaluated and reported on, such as the Oncokompas [25–29].

Remarkably, patients with chronic comorbid conditions needed more information on several subjects, but did *not* search for more information themselves, or consult a health care provider specifically for more information. Possibly, because these patients receive regular checks at their GP for their comorbid condition, they address any questions during those visits. However, it could also be that the information needs of patients with comorbid conditions are therefore not brought to the attention of health care providers and not adequately addressed. We have not come across this in previous literature, although Finney Rutten et al. did report that older patients seek information less frequently than younger patients [15], and we recommend all follow-up care providers to consider actively addressing these potential unmet needs. Perhaps the GP, who is familiar with treating comorbid conditions and trained in managing the complex interplay of medical history and personal background, could play an important role in reaching this potentially vulnerable group of patients. The percentage of patients with a chronic comorbid condition in our study seems comparatively low for the patient population. Although we combined information from patients themselves with hospital and general practice records, half of our participants did not appear to have any chronic comorbid condition. Perhaps, older patients with comorbid conditions were less inclined to participate in this study, but it is probably also a reflection of our strict definition of a “chronic” comorbid condition.

### Strengths and limitations

This paper is the first in the Netherlands to present both the information needs and information seeking behaviour reported by CRC patients currently in follow-up care. Furthermore, it provides an overview of CRC patients spread out over the entire follow-up care stage. The survey consisted of specific subjects, but also allowed patients to answer open questions in order to obtain a complete view of relevant issues. This allowed us to compare our patient population to previous publications, but also revealed new results.

Valid for the patients currently in a follow-up care program in the Netherlands, these results might not be readily applicable to the health care situation in other countries. Even though the spread of patients over time was acceptable, most patients were concentrated in the first year-and-a-half after treatment. Lastly, these results reflect patients’ perceptions on being informed; a patient who stated to be informed about a subject may quite possibly have inaccurate or erroneous knowledge.

### Conclusions

Colorectal cancer patients after initial treatment feel well informed about their treatment, disease, follow-up care program, and how to contact the hospital if necessary, but less informed about future expectations, nutrition, recommended physical activity, and heredity of cancer. Younger patients and patients who have undergone chemotherapy need more information and seek for more information themselves. Patients with comorbid conditions also need more information on several subjects, but do not seek for more information themselves. The GP is the most frequently consulted caregiver, which provides GPs especially with the opportunity to improve information provision on several subjects, while identifying and supporting vulnerable patients during their cancer follow-up stage.

#### Funding statement

This study was funded by Alpe d’HuZes, the Dutch Cancer Society (grant number UVA 2013-5954).

## References

[1] Faul LA, Rivers B, Shibata D, Townsend I, Cabrera P, Quinn GP, Jacobsen PB (2012). Survivorship care planning in colorectal cancer: feedback from survivors & providers. J Psychosoc Oncol.

[2] Rodriguez-Bigas MA, Chang GJ, Skibber JM (2007). Barriers to rehabilitation of colorectal cancer patients. J Surg Oncol.

[3] Husson O, Mols F, van de Poll-Franse LV (2011). The relation between information provision and health-related quality of life, anxiety and depression among cancer survivors: a systematic review. Ann Oncol.

[4] Brown S, Greenfield D, Thompson J (2016). Knowledge and awareness of long-term and late treatment consequences amongst colorectal cancer survivors: a qualitative study. EJON.

[5] Nijman J, Hendriks M, Brabers A, de Jong J, Rademakers J (2014). Patient activation and health literacy as predictors of health information use in a general sample of Dutch health care consumers. J Health Commun.

[6] Tan AS, Nagler RH, Hornik RC, DeMichele A (2015). Evolving information needs among colon, breast, and prostate cancer survivors: results from a longitudinal mixed-effects analysis. Canc Epidemiol Biomarkers Prev.

[7] van Mossel C, Leitz L, Scott S, Daudt H, Dennis D, Watson H (2012). Information needs across the colorectal cancer care continuum: scoping the literature. EJCC.

[8] Marijnen CAM. Oncoline. National guideline colorectalcarcinoma 2014. Last assessed 7th of January 2019 Oncoline2014. Available from: https://www.oncoline.nl/colorectaalcarcinoom. Accessed 7 Jan 2019.

[9] Kievit J, Schadé E, Ansink AC, de Bock GH, van Doorn JAM, Koning CCE, Mali WPTM, Mossink MH, Nortier JWR, Segaar R, Verbeek ALM, Wiggers T, Goppel MA, Gersons-Wolfensberger DCM, Health Council of the Netherlands (2007). Follow-up in oncology. Identify objectives, substantiate actions.

[10] Knottnerus JA, Wijffels, JFAM : SCK of KWF Kankerbestrijding. Dutch Cancer Society's Signalling Committee on Cancer. Aftercare in cancer; the role of primary care ; 2011. 2011.

[11] Eyck MAMF, Rosmalen, CFH, ter Brugge, A, Hogendorp, J, Romijn, EC, de Wit, NJ. The Dutch College of General Practitioners (NHG). Future prospective General Practitioners care 2022. https://issuu.com/lhvhuisartsen/docs/toekomstvisiehuisartsenzorg2022: 2012. Accessed 7 Jan 2019.

[12] Rubin G, Berendsen A, Crawford SM, Dommett R, Earle C, Emery J, Fahey T, Grassi L, Grunfeld E, Gupta S, Hamilton W, Hiom S, Hunter D, Lyratzopoulos G, Macleod U, Mason R, Mitchell G, Neal RD, Peake M, Roland M, Seifert B, Sisler J, Sussman J, Taplin S, Vedsted P, Voruganti T, Walter F, Wardle J, Watson E, Weller D, Wender R, Whelan J, Whitlock J, Wilkinson C, de Wit N, Zimmermann C (2015). The expanding role of primary care in cancer control. Lancet Oncol.

[13] Salz T, Baxi SS, Blinder VS, Elkin EB, Kemeny MM, McCabe MS (2014). Colorectal cancer survivors' needs and preferences for survivorship information. J Oncol Pract.

[14] Health Information National Trends Survey (HINTS) - Part of NCI's Division of Cancer Control and Population Sciences: NIH National Cancer Institute; 2019 [07-05-2019]. Available from: https://hints.cancer.gov/publications-reports/hints-publications.aspx. Accessed 7 May 2019.

[15] Finney Rutten LJ, Agunwamba AA, Wilson P, Chawla N, Vieux S, Blanch-Hartigan D, Arora NK, Blake K, Hesse BW (2016). Cancer-related information seeking among cancer survivors: trends over a decade (2003-2013). J Cancer Educ.

[16] Averyt JC, Nishimoto PW (2014). Psychosocial issues in colorectal cancer survivorship: the top ten questions patients may not be asking. JCO.

[17] Wright SJ, Gibson D, Eden M, Lal S, Todd C, Ness A, Burden S (2017). What are colorectal cancer survivors' preferences for dietary advice? A best-worst discrete choice experiment. J Cancer Surviv.

[18] Rutten LJ, Arora NK, Bakos AD, Aziz N, Rowland J (2005). Information needs and sources of information among cancer patients: a systematic review of research (1980-2003). Patient Educ Couns.

[19] Doherty Meredith, Miller-Sonet Ellen, Gardner Daniel, Epstein Irwin (2018). Exploring the role of psychosocial care in value-based oncology: Results from a survey of 3000 cancer patients and survivors. Journal of Psychosocial Oncology.

[20] Bours MJ, Beijer S, Winkels RM, van Duijnhoven FJ, Mols F, Breedveld-Peters JJ, Kampman E, Weijenberg MP, van de Poll-Franse LV (2015). Dietary changes and dietary supplement use, and underlying motives for these habits reported by colorectal cancer survivors of the Patient Reported Outcomes Following Initial Treatment and Long-Term Evaluation of Survivorship (PROFILES) registry. Br J Nutr.

[21] Chawla N, Blanch-Hartigan D, Virgo KS, Ekwueme DU, Han X, Forsythe L, Rodriguez J, McNeel TS, Yabroff KR (2016). Quality of Patient-Provider Communication Among Cancer Survivors: Findings From a Nationally Representative Sample. J Oncol Pract.

[22] Cumbo A, Agre P, Dougherty J, Callery M, Tetzlaff L, Pirone J, Tallia R (2002). Online cancer patient education: evaluating usability and content. Cancer Pract.

[23] Sajid MS, Iftikhar M, Monteiro RS, Miles AF, Woods WG, Baig MK (2008). Internet information on colorectal cancer: commercialization and lack of quality control. Colorectal Dis.

[24] Okuhara T, Ishikawa H, Urakubo A, Hayakawa M, Yamaki C, Takayama T, Kiuchi T (2018). Cancer information needs according to cancer type: a content analysis of data from Japan's largest cancer information website. Prev Med Rep.

[25] Jansen F, van Uden-Kraan CF, van Zwieten V, Witte BI, Verdonck-de Leeuw IM (2015). Cancer survivors' perceived need for supportive care and their attitude towards self-management and eHealth. Support Care Cancer.

[26] Duman-Lubberding S, van Uden-Kraan CF, Jansen F, Witte BI, van der Velden LA, Lacko M, Cuijpers P, Leemans CR, Verdonck-de Leeuw IM (2016). Feasibility of an eHealth application "OncoKompas" to improve personalized survivorship cancer care. Support Care Cancer.

[27] Duineveld LA, Wieldraaijer T, Asselt KM v, Nugteren IC, Donkervoort SC, Ven AW vd, Smits AB, van Geloven AAW, Bemelman WA, Beverdam FH, van Tets WF, Govaert MJPM, Bosmans JE, Verdonck-de Leeuw IM, Uden-Kraan CF, Weert van HCPM, Wind J. Improving care after colon cancer treatment in the Netherlands, personalised care to enhance quality of life: study protocol for a randomised controlled trial (I CARE study). Trials. 2015;16:284. 10.1186/s13063-015-0798-7. 10.1186/s13063-015-0798-7 PMC4499213 26112050

[28] Corbett T, Singh K, Payne L, Bradbury K, Foster C, Watson E, Richardson A, Little P, Yardley L (2018). Understanding acceptability of and engagement with Web-based interventions aiming to improve quality of life in cancer survivors: a synthesis of current research. Psycho-oncology.

[29] Verdonck-de Leeuw IM. OncoKompas 2.0 2019 [23-01-2019]. Available from: https://www.oncokompas.nl/. Accessed 23 Jan 2019.

